# Humeral Capitellum Fractures in Adolescents: A Study of 6 Cases Treated by Open Reduction and Internal Fixation with Bioabsorbable Nails

**DOI:** 10.1155/2022/4012125

**Published:** 2022-04-11

**Authors:** Łukasz Wiktor, Ryszard Tomaszewski

**Affiliations:** ^1^Upper Silesian Children's Health Centre, Department of Trauma and Orthopaedic Surgery, Katowice, Poland; ^2^Faculty of Science and Technology, Institute of Biomedical Engineering, University of Silesia in Katowice, Katowice, Poland

## Abstract

**Purpose:**

The purpose of our study was to evaluate the clinical outcome following open reduction and internal fixation of humeral capitellum fractures in adolescents and to assess the usefulness of bioresorbable implants in that procedure. Due to the rarity of these fractures, there are not many studies dealing with the problem in the literature.

**Methods:**

We retrospectively evaluated a group of 6 skeletally immature patients aged 10.6–15.3 treated at our department from January 2015 to December 2021. Four type I and two type IV were diagnosed based on the Bryan and Morrey classification. Our patients underwent an open reduction and internal fixation of coronal shear fractures with the use of SmartNail®.

**Results:**

All patients were satisfied with the treatment outcome and had full pronation and flexion after surgery. Two patients presented minor deficits of extension and supination compared with the contralateral elbow. At the one-year follow-up, all patients scored 100 on the Mayo Elbow Performance Score.

**Conclusions:**

Correct diagnosis and early surgical intervention in humeral capitellum fractures are crucial. That fractures should be anatomically reduced with no articular cartilage damage in order to prevent osteoarthritis. Based on our experience, SmartNail® implant is accurate for the osteochondral fragment fixation.

## 1. Introduction

Fractures of the humeral capitellum are rare and account for 1% of elbow fractures [1–4]. Moreover, coronal shear fractures are rare in children under twelve due to the cartilaginous structure of the distal humerus [5–7]. In children, traumatic shear forces acting on the distal humeral epiphysis more often cause lateral condyle fracture. As the humerus ossifies, the risk of capitellum fracture increases [6]. The most popular classification scale of distal humeral shear fractures is the Bryan and Morrey classification [8], which divides fractures into the four types: type I (known as Hahn–Steinthal fracture) is a osteochondral fracture which involves the capitellum with or without small fragment of trochlea, type 2 (called the Kocher–Lorenz fracture) is a capitellum fracture containing the cartilaginous cap with a small amount of subchondral bone, type 3 is a comminuted fracture of the capitellum, and type 4 (added by McKee et al.) [9] defining an osteochondral fracture of the capitellum with a significant fragment of a trochlea. Coronal plane articular fractures of the distal humerus can be missed, especially based on anteroposterior radiographs [10, 11]. True lateral X-rays are most helpful for diagnosis [12, 13]. The symptom of a double arch is described in the literature, which is typical for type IV fractures according to the Bryan and Morrey classification [8]. CT scan is crucial for the correct diagnosis [14]. The typical clinical symptoms include swelling, pain, and limited active and passive elbow motions. Due to the rarity of humeral capitellum fractures in the skeletally immature population, there are only a few studies dealing with the mentioned problem in the literature. Because bioresorbable polymer implants are rapidly growing alternatives to traditional implants, especially in children, our aim was to evaluate the usefulness of these implants in treatment of the abovementioned fractures [15].

## 2. Materials and Methods

**Figure 1:**
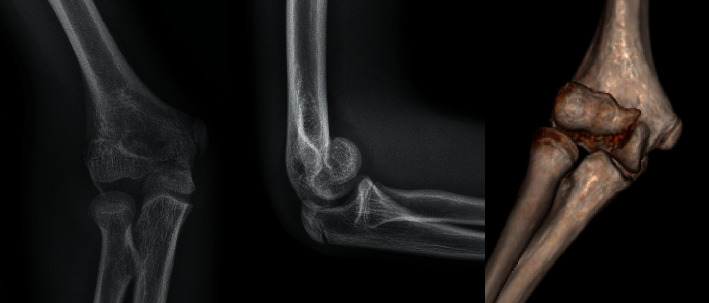
Patient number 4, preoperative X-rays, and CT scan. Type IV/McKee fracture according to Bryan and Morrey classification. Double arch sign is present on the lateral view.

**Figure 2:**
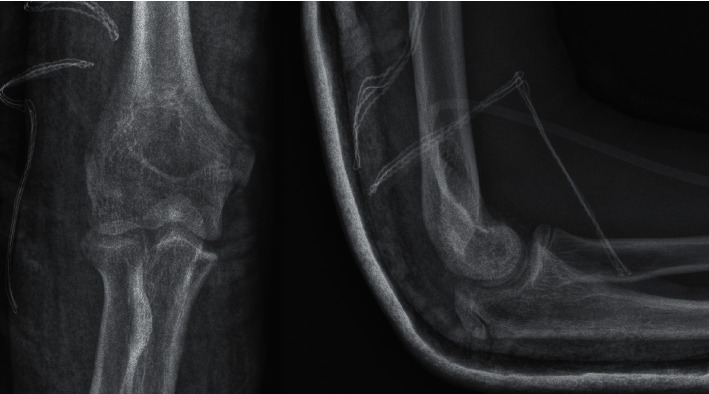
Patient number 4, postoperative X-rays. Osteochondral fragment reduced and fixated with 3 × SmartNail.

After retrospective medical charts review of all patients with elbow fractures, we enrolled into the study group only the cases with humeral capitellum fractures. From January 2015 to December 2021, 6 patients with a capitellum fracture were treated at our institution, which represents only 0.3% of all elbow fractures seen in the same period. All coronal plane articular humeral fractures were treated with open reduction and internal fixation (ORIF) with the use of SmartNail (ConMed Linvatec Ltd.), which are osteochondral fixation nails made from bioabsorbable PLA copolymer [16, 17]. All patients were diagnosed based on elbow X-rays in anteroposterior and lateral projections. A CT scan was obtained in all patients to further characterize the fracture and for proper surgical planning. An illustrative case is shown in Figure 1. Type I fracture was assigned to four patients and type IV to the remaining two patients. Four patients sustained injury after falling on an outstretched hand and two fell on flexed elbows. An overview of the study group is given in Table 1. Based on measurements on the CT scans, we chose the proper length of bioresorbable pins (1.50 mm × 20.00 mm nails only). We always performed the surgery with a tourniquet and with the patient in a supine position. Each time, we used the lateral approach with skin incision centered over the lateral epicondyle. We kept the forearm pronated to protect the radial nerve, which runs close to the radial head and divides it into its superficial and deep branches over the radial head. After incising the deep fascia, we elevated the muscles and capsule subperiosteally and split the extensor muscles anterior to the lateral collateral ligament. The fracture was debrided of haematoma, and saline irrigation was applied. Osteochondral fragments were replaced and temporarily stabilised with K-wires. Final fixation was made with SmartNail in an anteroposterior direction after the direct visual and intraoperative X-ray control. SmartNail' heads were always slightly buried beneath the articular surface to avoid impingement and further osteoarthritis. Then, definitive reduction extensors were repaired to the cuff on the lateral supracondylar ridge. The surgery elbow was immobilised in a cast at a right angle for two weeks until the first outpatient control. An example of the postoperative radiographs from patient number 4 is shown in Figure 2. Patients were followed up with 6 and 12 weeks postoperatively to evaluate rehabilitation progress and if need to modify the exercise program. The final follow-up period was one year. Flexion-extension and pronation-supination movements were compared with the contralateral side. Pain intensity, range of motion, stability, and elbow function were evaluated according to the Mayo Elbow Performance Score [18].

## 3. Results

6 patients treated in our institute were analyzed (four boys and two girls). Average age was 13.2 (10.6–15.3), average height was 145.6 cm (130–163), average weight was 49 kg (35–60), and average BMI was 22.9 (20.7–25.5).

The right and left humerus were equally involved in three cases. Four fractures were classified as type I and two as type IV according to the Bryan and Morrey classification.

At the first-year follow-up, our patients presented no pain and excellent elbow function. All patients scored one hundred on the Mayo Elbow Performance Score. All patients had full pronation and flexion. Two patients presented minor deficits of extension and supination compared with the contralateral elbow.  Table 2 provides operated elbow range of motion in relation to the healthy side. Postoperative X-rays did not reveal any nonunions and humeral avascular necrosis. Furthermore, we did not observe elbow angular deformity and any case of physeal arrest.

## 4. Discussion

**Figure 3:**
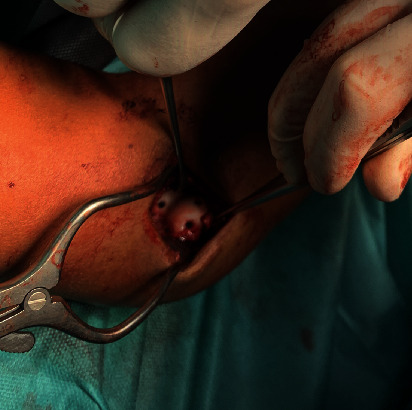
Patient number 4, intraoperative view after definitive fixation (2 × SmartNail in the capitellum and 1 × SmartNail in the trochlea).

**Figure 4:**
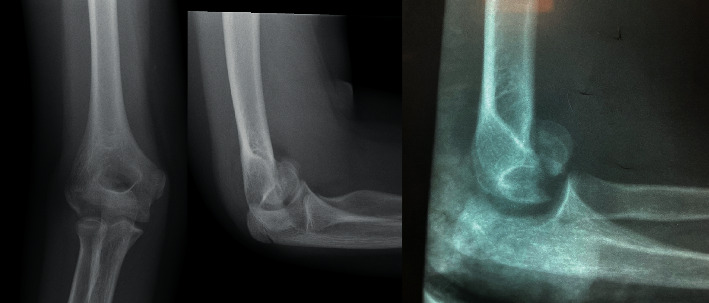
Patient number 1, posttraumatic X-rays, and control lateral view after 3 weeks from injury.

Because bioresorbable polymer implants are rapidly growing alternatives to traditional implants, especially in children, our aim was to evaluate the usefulness of these implants in treatment shear fractures of the distal humerus in the skeletally immature population. The SmartNail® implant is a bioabsorbable bone fixation nail designed for the osteochondral fragments fixation resulting from trauma or osteochondritis dissecans lesions. The surgical technique in which the osteochondral fragments are stabilised with biodegradable pins allows the omission of metal implants insertion which in our opinion is important in intraarticular fractures. Moreover, SmartNail implant biodegrades in vivo for 2-3 years. Reducing the number of elbow surgical procedures, including implants removal, always corresponds with the better final result. It also reduces the stress associated with subsequent surgeries which in our opinion is beneficial in children. Nails have appropriate initial mechanical strength and stiffness, which gives the possibility of adequate fracture stabilization. The protrusions on the nail body allow for a stable implant anchoring in the humeral epiphysis. Based on our study, open reduction and internal fixation with placement of two or three nails allow the achievement of precise compression and accurate stability (Figure 3). In the study group, based on the osteochondral size, we stabilized the type IV fracture with three pins and the type I fracture with two pins. Sufficient fixation checked during the surgery allowed the patient to start early rehabilitation after cast removal, which was obligatory in our group two weeks after the operation. Short cast immobilization and early elbow mobilisation corresponds with the short reconvalescence period and regaining of a full range of motion. In the study group, two patients were admitted to our department with delay. Patient number 1 was misdiagnosed due to the low quality of posttraumatic radiological examinations (Figure 4). Patient number 2, despite restricted elbow motion, was referred later because of a minor pain. We are of the opinion that prolonged cast immobilization in those cases caused extension and supination deficits as given in Table 2. There are only a few studies describing shear fractures of the distal humerus in the skeletally immature population. There is no consent in literature about the optimal surgical technique and internal fixation [15, 19]. Because of that, this medical issue is challenging. Excision of the fragment is forbidden in children because of the elevated risk of osteoarthritis. The closed reduction stays historical and currently is not applicable due to the intraarticular nature and necessity for anatomical reposition. Several studies describe stabilization of the fractures with K-wires, but this does not provide adequate stability. Moreover, the need for long cast immobilization brings the risk of motions deficit [5, 13]. The complex nature of these injuries constitutes a challenge for looking at the optimal fixation techniques. The literature contains reports describing stabilization of osteochondral humerus fractures with the use of Herbert screws, which are the headless double-threaded screws [13, 19–25]. Herbert screws allow proper fracture compression due to its double threads. Moreover, lack of head allows for burial beneath the articular surface [26]. Our patients treated with bioabsorbable fixation nails achieved excellent surgical outcomes. All patients one year after surgery scored one hundred on the Mayo Elbow Performance Score. Dubberley et al., in their group of twenty-eight patients, obtained an average score on the Mayo Elbow Performance Index (91 ± 11) [27]. In our opinion, this difference is because our group consisted of skeletally immature patients, which corresponds with better outcomes. Avascular necrosis is not common after ORIF procedures [9]. Mahirogullari et al. report 0–30% incidence of avascular necrosis [12]. We did not observe such a complication in our study group. No patient in our series had physeal arrest or angular deformity. Based on our experience, we highly recommend open reduction and internal fixation with bioabsorbable SmartNail to treat humeral capitellum fractures in adolescent age groups.

## 5. Conclusions

Correct diagnosis and early surgical intervention in humeral capitellum fractures are crucial. That fractures should be anatomically reduced with no articular cartilage damage in order to prevent osteoarthritis. Based on our experience, SmartNail® implant is accurate for the osteochondral fragment fixation.

## Figures and Tables

**Table 1:** The study group overview.

No.	Age (years)	Side	Bryan and Morrey classification	AO classification	Mechanism of injury	Surgical technique
1	12.5	Right/dominant	Type IV/McKee	AO 13B3.3	Falling on an outstretched hand/standing height	ORIF/3 × SmartNail
2	14.3	Left/dominant	Type I/Hahn–Steinthal	AO 13B3.1	Falling on a flexed elbow/bicycle	ORIF/2 × SmartNail
3	13.2	Right/dominant	Type I/Hahn–Steinthal	AO 13B3.1	Falling on an outstretched hand/2 meters high	ORIF/2 × SmartNail
4	15.3	Right/dominant	Type IV/McKee	AO 13B3.3	Falling on a flexed elbow/scooter	ORIF/3 × SmartNail
5	10.6	Left/nondominant	Type I/Hahn–Steinthal	AO 13B3.1	Falling on an outstretched hand/standing height	ORIF/2 × SmartNail
6	13.4	Left/nondominant	Type I/Hahn–Steinthal	AO 13B3.1	Falling on an outstretched hand/standing height	ORIF/2 × SmartNail

**Table 2:** Operated elbow range of motion in relation to the healthy side.

No.	Bryan and Morrey classification/affected side	Healthy side, ROM (degrees)	Affected side, ROM deficits (degrees)	Time from injury to surgery (days)
1	Type IV/right side	Ext/flex: −5/130° Pro/sup: 90/90°	Deficit of extension: 10° Deficit of supination: 5°	20
2	Type I/left side	Ext/flex: 0/140° Pro/sup: 90/90°	Deficit of extension: 10° Deficit of supination: 10°	28
3	Type I/right side	Ext/flex: 0/140° Pro/sup: 80/90°	No deficits	0
4	Type IV/right side	Ext/flex: 0/140° Pro/sup: 90/90°	No deficits	0
5	Type I/left side	Ext/flex: −10/150° Pro/sup: 90/90°	No deficits	0
6	Type I/left side	Ext/flex: 0/140° Pro/sup: 80/90°	No deficits	2

## Data Availability

The data used to support the results of this study are available at Upper Silesian Children's Health Centre, Department of Trauma and Orthopaedic Surgery, Katowice, Poland.
